# Residual Viremia Is Preceding Viral Blips and Persistent Low-Level Viremia in Treated HIV-1 Patients

**DOI:** 10.1371/journal.pone.0110749

**Published:** 2014-10-29

**Authors:** Laura Marije Hofstra, Tania Mudrikova, Arjen J. Stam, Sigrid Otto, Kiki Tesselaar, Monique Nijhuis, Annemarie M. J. Wensing

**Affiliations:** 1 Department of Medical Microbiology, Virology, University Medical Center Utrecht, Utrecht, the Netherlands; 2 Department of Internal Medicine and Infectious Diseases, University Medical Center Utrecht, Utrecht, the Netherlands; 3 Laboratory of Translational Immunology, University Medical Center Utrecht, Utrecht, the Netherlands; Centro Nacional de Microbiología - Instituto de Salud Carlos III, Spain

## Abstract

**Background:**

It has been suggested that low-level viremia or blips in HIV-infected patients on antiretroviral treatment are related to assay variation and/or increased sensitivity of new commercial assays. The 50-copy cut-off for virologic failure is, therefore, under debate.

**Methods:**

Treated patients with low-level viremia (persistent viral loads (VL) of 50–1000 copies/mL, group A, N = 16) or a blip (single detectable VL, group B, N = 77) were compared to a control group (consistently suppressed viremia since start therapy (<50 copies/mL), N = 79). Residual viremia (detectable viral RNA <50 copies/ml) in the year preceding the first VL above 50 copies/mL (T0) was determined using Roche Cobas-Amplicor v1.5 or CAP-CTM v2.0. Subsequent virologic failure (2 consecutive VLs>500 or 1 VL>1000 copies/mL that was not followed by a VL<50 copies/mL; median follow up 34 months) was assessed.

**Results:**

Significantly more patients in groups A and B had residual viremia in the year preceding T0 compared to controls (50% and 19% vs 3% respectively; p<0.001). Residual viremia was associated with development of low-level viremia or blips (OR 10.9 (95% CI 2.9–40.6)). Subsequent virologic failure was seen more often in group A (3/16) and B (2/77) than in the control group (0/79).

**Conclusion:**

Residual viremia is associated with development of blips and low-level viremia. Virologic failure occurred more often in patients with low-level viremia. These results suggest that low-level viremia results from viral production/replication rather than only assay variation.

## Introduction

Combination antiretroviral therapy (cART) suppresses HIV replication, resulting in a decline of the plasma viral load (VL). The goal of cART historically followed the limit of detection of the assay used to measure HIV RNA levels. Currently in clinical practice the cut-off of 50 copies per milliliter is used [1], [2] but after the introduction of new commercial assays with increased sensitivity and a limit of detection below 50 copies/mL this cut-off has become subject of debate.

Viremia below 50 copies/mL is often referred to as residual viremia. After initiation of cART the VL usually declines below the established cut-off of 50 copies within 3–6 months and continues to decline further within the first year. In clinical practice, a substantial number of patients achieve maximal suppression. In these patients the assay cannot detect any signal, referred to as target not detected (TND). However, in a selection of patients on cART persistent or transient residual viremia below the cut-off of 50 copies/mL is observed [3], [4]. It is a matter of longstanding debate whether residual viremia is a result of virus production by latently infected cells or is caused by ongoing viral replication despite therapy with the risk of selection of resistance. Therefore its clinical relevance remains uncertain. In a cross sectional analysis of patients on cART, Doyle *et al* showed that residual viremia enhances the chance of viral rebound (viremia above 50 copies/mL) and therefore suggested that the goal of cART may need to be revised to a lower cut-off than 50 copies/mL [5].

Viral rebound can be either transient (a single viral load above 50 copies/mL), generally referred to as viral blip, or persistent (continuous detectable viral load between 50 and 1000 copies/mL) which is called low-level viremia. As for residual viremia, there is vivid discussion regarding the source and clinical relevance of viral blips and low-level viremia.

After introduction of the Roche Cobas Taqman assay, which has a limit of detection of 20 copies/mL and a higher rate of detectability than the former Roche Amplicor assay [6], physicians worldwide observed an increase in the frequency of blips [7], [8]. This mounted the idea that this was a result of assay variation and/or increased sensitivity rather than virus production or replication [9], [10]. Based on these discussions the DHHS Guidelines for use of antiretroviral agents raised the definition of virologic failure to a confirmed viral load above 200 copies/mL, assigning most cases of apparent viremia to isolated blips or assay variability with no increased risk of virologic failure [11].

This proposed cut-off of 200 copies/mL is not based on extensive clinical data. Several studies have looked in to the risk of virologic failure after viral rebound, but data on viral rebound between 50 and 200 copies/mL is limited. A large observational cohort of patients on cART recently showed that even low-level viremia between 50 and 199 copies/mL was associated with increased risk of virologic failure [12]. Furthermore, it was recently shown that levels of activated (CD3+ HLA-DR+) T cells predicted the occurrence of viral blips, suggesting viral rebound may reflect viral production from activated immune cells instead of assay variation [13]. An association between immune activation and a modest increased risk of a subsequent blip was also observed using CD38/HLA-DR expression on CD8+ T cells [14].

To investigate the relevance of blips and low-level viremia, we compared patients with and without blips and low-level viremia from our clinical center: we determined the presence of residual viremia in the year preceding viral rebound, assessed a possible role for immune activation and studied rates of subsequent virologic failure.

## Methods

### Ethical statement

Patients were included from the observational AIDS Therapy Evaluation in the Netherlands (ATHENA) cohort, which follows HIV-positive patients who are registered in one of the designated treatment centers in the Netherlands. Patients can opt-out after being informed on the purpose of data collection by their treating physician. Patients who have decided not to opt out are anonymously recorded in a central database.

### Patients

**Figure 1:**
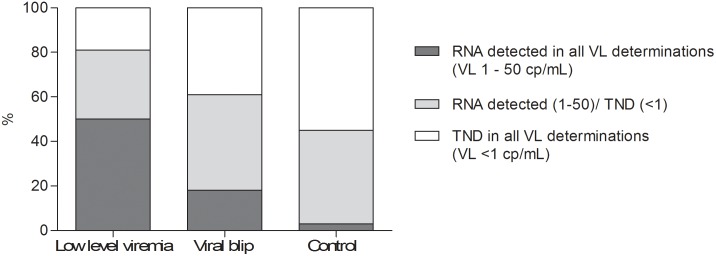
Methods. A  =  patients with low-level viremia; B  =  patients with a single viral blip; C  =  patients with continuously suppressed viremia; T0 =  first detectable viral load for patients of group A and B. Analysis of viral loads in year preceding T0 to determine level of residual viremia. Follow up to determine rate of subsequent virologic failure.

Adult HIV-infected patients who had a first detectable VL (T0) in the period April 2009– August 2010 were identified retrospectively from our outpatient clinic (University Medical Center Utrecht) to allow two years of follow-up. Patients treated with cART for more than 1 year who achieved at least 2 consecutive VL<50 copies/mL were included. Patients who had documented treatment interruption were excluded. Patients were grouped according to their VL pattern (figure 1). Group A included patients with repeatedly consecutive detectable VL (e.g. T0 was followed by more samples with a detectable VL, with a maximum plasma viral load of 1000 copies/mL; thus considered to have low-level viremia) and group B included patients with a single detectable VL (e.g. T0 was followed by a sample with an undetectable VL without a change in treatment; which we considered a viral blip). Both groups were compared to a control group of randomly selected adult HIV-infected patients who also were treated with cART for at least 1 year and did not experience detectable viremia after achieving viral suppression. For these patients, T0 was defined as the VL determination closest to the mean date of T0 in group A and B, allowing analysis of a similar timeframe.

### Data collection

Clinical data were retrieved from the local Athena database. To determine the role of residual viremia, a longitudinal analysis of VL results below 50 copies/mL in the year preceding T0 was performed. VL monitoring was performed on EDTA plasma samples using Roche Cobas Taqman v2.0. Part of the VL determinations in the year preceding T0 were performed with the Roche Cobas Amplicor v1.5 assay, which was a similar percentage among groups. The result of the VL determination was either RNA detected (e.g. a VL between 1 and 50 copies/mL, residual viremia) or target not detected (TND, e.g. no signal in the PCR, zero viremia). We determined the percentage of patients who had in the year preceding T0 (1) RNA detected at all determinations, (2) TND at all determinations or (3) RNA detected in some determinations and TND in others.

### Follow-up

All patients were followed up from T0 for at least 24 months to determine whether virologic failure or a subsequent episode of detectable viremia occurred. Virologic failure was defined as two consecutive VLs >500 copies/mL or a VL exceeding 1000 copies/mL that was not followed by a VL<50 copies/mL on the same regimen. A secondary outcome was a subsequent episode of detectable viremia (VL>50 copies/mL). Patients with low-level viremia first had to achieve a VL<50 copies/mL before they were classified as having a subsequent episode of detectable viremia. Population genotypic analysis was performed if requested by treating physician. Resistance-associated mutations were defined according to the IAS-USA mutation list [15].

### Immune activation markers

In a random subset of patients, stored samples of T0 were analyzed for levels of immune activation markers. The concentration of soluble markers CD14 and CXCL9 was measured using ELISAs. All samples were assayed on the same plate.

### Statistical analysis

Baseline clinical laboratory and treatment characteristics were compared between group A, B and control group using chi-squared, one way ANOVA and Kruskal-Wallis tests. A logistic regression model was used to identify factors that were associated with the occurrence of blips and low-level viremia. Residual viremia in the year preceding T0 and the duration of treatment were included in the multivariable analysis, as well as all other factors with a p value <0.2 in univariable analysis. Factors with a p value<0.05 in multivariable analysis were considered to be associated with blips and low-level viremia. Standard Kaplan-Meier estimation and log-rank comparisons were used to evaluate time to virologic failure and time to detectable viremia. Due to a very low number of events Cox regression models for assessing factors associated with virologic failure could not be used. All of the statistical tests were two sided at the 5% level and performed with SPSS software (version 20.0).

## Results

In total, 93 eligible patients were identified and included in the study group; 16 patients with low-level viremia (group A) and 77 patients with a viral blip (group B). For group A, the maximum level of low-level viremia was 50–200 copies/mL for 10 patients, 200–500 copies/mL for 4 patients and 500–1000 copies/mL for 2 patients. For group B, 53 patients had a blip between 50 and 200 copies/mL, 14 patients between 200–500 copies/mL and 10 patients >500 copies/mL. 79 patients were assigned to the control group. In all groups, the majority was male, of European origin with a mean age around 45 years (table 1). No differences in CD4 count at presentation were observed. There was a trend towards shorter duration of cART and more PI-based regimens in group A, compared to group B and the control group.

**Table 1:** Baseline characteristics of the study population.

Characteristic	Study group		Control group	P
	Group A(LLV)	Group B(blip)		
*N*	16	77	79	
Age – yr	45±11	46±9	45±11	0.77
Male sex	11 (69)	61 (79)	61 (77)	0.66
Origin				0.84
*European*	11 (69)	51 (66)	56 (71)	
*Sub Saharan African*	3 (19)	17 (22)	15 (19)	
*Other*	2 (12)	9 (12)	8 (10)	
Route of transmission				0.21
*MSM*	9 (56)	31 (40)	31 (39)	
*Heterosexual*	5 (31)	33 (43)	25 (32)	
*Other*	2 (12)	13 (17)	23 (29)	
Duration of known HIV infection – months	83±52	116±75	104±63	0.33
Baseline CD4 count – median(Q1, Q3)	285(109, 460)	266(89, 443)	308(146, 471)	0.85
Baseline log viral load – median(Q1, Q3)	5.1(3.9, 6.3)	5.3(4.8, 5.8)	5.2(4.7, 5.7)	0.56
Duration of cART – months	47±39	72±50	74±50	0.06
cART regimen				0.10
*NNRTI based*	5 (31)	36 (47)	48 (61)	
*PI based*	11 (69)	37 (48)	29 (37)	
Time to next visit				<0.001
*1–2 months*	9 (56)	47 (61)	2 (3)	
*3 months or more*	7 (44)	30 (39)	77 (98)	

**Figure 2:**
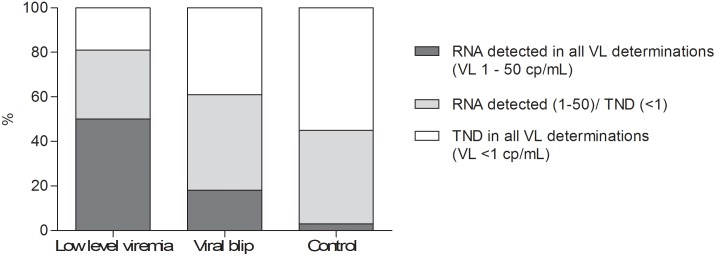
Longitudinal analysis of viral load results in preceding year. Low-level viremia is often preceded by positive VL results; in 50% of these patients HIV-1 RNA was detected in all VL determinations in the preceding year, compared to only 3% of patients with a sustained suppressed VL (p<.0001).

We analyzed the presence of residual viremia in the year preceding T0 (figure 2). In all three groups the number of viral load determinations with a result below 50 copies/mL was comparable, with a median number of samples of 2 in group A (range: 2–5), 3 in group B (range 2–6) and 3 in the control group (range 2–5). Significantly more patients in group A and B (50% and 19%) had residual viremia in all VL determinations in the year preceding T0, compared to the control group (3%; p<0.0005). The majority of patients (55%) in the control group had no RNA detected (target not detected  = TND) in all VL determinations. Patients with continuous residual viremia had a significantly shorter treatment duration compared to patients with continuous TND (mean 41 vs. 83 months, p<0.001).

Correlates of detectable viremia were determined with use of a logistic regression model (table 2). In univariable analysis, heterosexual transmission route, PI-based regimen and detected residual viremia in the preceding year showed an increased odds ratio for developing detectable viremia and were included in multivariable analysis. Only residual viremia in the preceding year was associated with the development of detectable viremia in multivariable analysis (OR 10.9 (95% CI 2.9–40.6)).

**Table 2:** Logistic Regression Analysis of correlates of detectable viremia>50 copies/mL at T0, multivariable analysis.

	Univariable analysis		Multivariable analysis	
	OR (95% CI)	P value	OR (95% CI)	P value
Age	1.01 (0.98–1.04)	0.51	-	-
Sex				
*Female*	1.00 (reference)			
*Male*	0.99 (0.48–2.02)	0.98	-	-
Origin				
*European*	1.00 (reference)			
*Sub Saharan African*	1.20 (0.56–2.58)	0.63	-	-
*Other*	1.24 (0.47–3.31)	0.67	-	-
Transmission route				
*MSM*	1.00 (reference)		1.00 (reference)	
*Hetero*	1.18 (0.59–2.35)	0.64	1.38 (0.66–2.85)	0.39
*Other*	0.51 (0.23–1.13)	0.10	0.48 (0.20–1.12)	0.09
Known HIV infection	1.01 (0.66–1.54)	0.98	-	-
Baseline (log) CD4 count	0.97 (0.77–1.22)	0.78	-	-
Baseline (log) viral load	0.96 (0.67–1.37)	0.82	-	-
Duration cART	0.83 (0.56–1.24)	0.37	1.25 (0.79–1.98)	0.35
Regimen				
*PI based*	1.00 (reference)		1.00 (reference)	
*NNRTI based*	0.52 (0.28–0.96)	0.04	0.64 (0.33–1.26)	0.21
*Other*	1.21 (0.21–7.02)	0.83	1.36 (0.22–8.55)	0.82
Residual viremia				
*Always TND*	1.00 (reference)		1.00 (reference)	
*TND/+*	1.50 (0.78–2.88)	0.22	1.74 (0.89–3.42)	0.11
*Always +*	9.56 (2.63–34.67)	0.001	10.90 (2.93–40.57)	<0.0005

In general, patients attend the outpatient clinic every 3–6 months, as was observed in patients of the control group (table 1, time to next visit: 97% 3 months or more). In contrast, the majority of patients in group A and B returned within the first 2 months after detectable viremia, before the regular scheduled visit.

**Figure 3:**
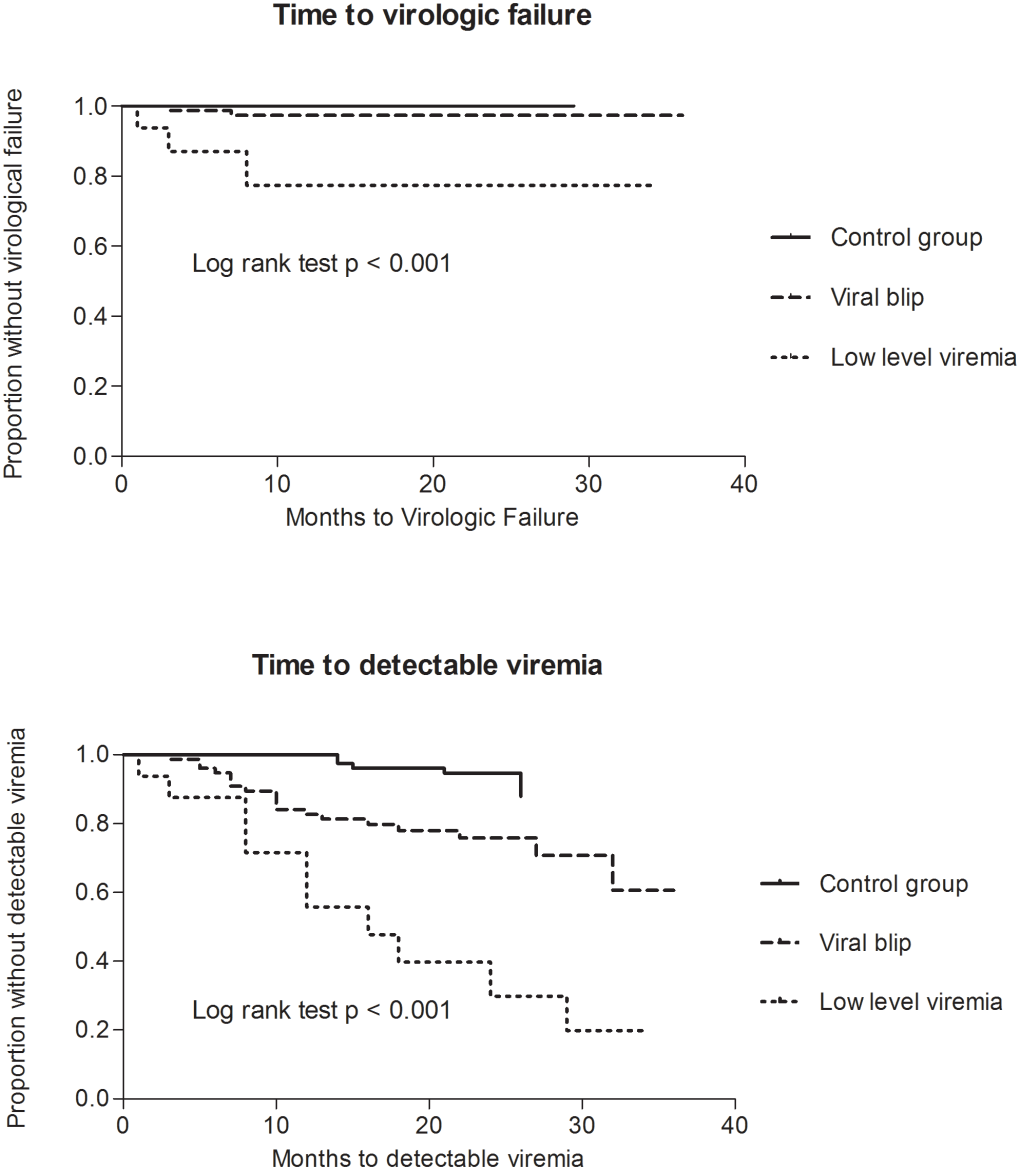
Kaplan-Meier plot of time to virologic failure (VL>1000 cp/mL) and of time to detectable viremia (≥50 cp/mL).

Patients were followed for a median of 34 months to determine the occurrence of virologic failure or a new episode of detectable viremia. Seven patients (3 of group B; 4 of control group) were lost to follow up. Virologic failure was observed in 3 patients of group A, 2 patients of group B and in none of the control group patients (figure 3). In the five patients with virologic failure, the first detectable viral load (T0) varied between 117 and 567 copies/mL. Considering the secondary outcome, detectable viremia was significantly more often seen in patients who previously had a viral blip or low-level viremia than in patients who had a sustained suppressed viral load. In the control group only 3 patients developed a first viral blip to levels below 200 copies/mL; all others maintained a suppressed VL.

In group A, all 3 patients with virologic failure were on a PI-based regimen. In one patient, adherence counseling without a change in therapy was sufficient to suppress the VL. For the other two, virologic suppression followed after a switch in therapy which was guided by drug resistance testing. Selection of multiple major PI and RT drug resistance mutations was observed in one of them. In 7 patients from group A without virologic failure resistance testing did not reveal resistance mutations relevant to the used regimen. Maintenance of the same regimen (6 PI-based, 1 NNRTI-based) – with an increased dosing of the boosted PI in 1 patient – resulted in sustained virologic suppression in five patients. Two patients switched therapy from an NNRTI-based to a PI-based regimen without guidance of a resistance test which was followed by virologic suppression in 1 patient.

In group B, two patients experienced virologic failure. Resistance testing did not reveal selection of new relevant major resistance mutations. Suppression on the same regimen (1 NNRTI-based, 1 PI-based) was achieved in both after adherence counseling. Resistance testing was also performed in three patients without virologic failure (2 NNRTI-based, 1 PI-based regimen) and did not show selection of resistance. One patient of group B developed an episode of low-level viremia after the initial blip and reached virologic suppression following a switch from an NNRTI to a PI-based regimen.

**Figure 4:**
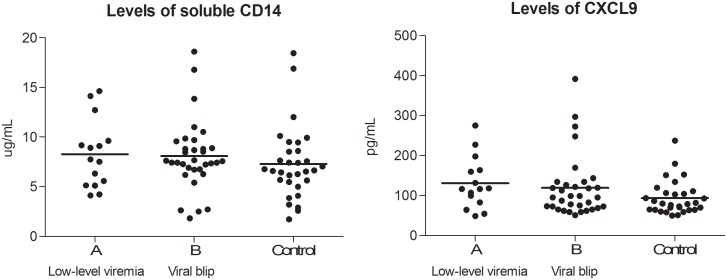
Levels of immune activation markers at T0. Lines indicate mean values. No significant difference in levels of soluble CD14 among the groups (p 0.489). There is a trend towards higher levels of CXCL9 in patients with low-level viremia and viral blips than in patients with continuously suppressed viremia (p 0.098).

To investigate a possible relation between immune activation and the occurrence of viral rebound, two soluble immune activation markers at T0 were studied in a random subset of patients (group A n = 15; group B n = 33; control group n = 30). A trend towards higher CXCL9 levels in patients with low-level viremia (mean 130,98 pg/mL) or a viral blip (mean 119,21) compared to patients with a consistently suppressed VL (mean 93,62 pg/mL; p 0.098). There was no difference in soluble CD14 levels among groups (Figure 4). We did not observe a relation between the levels of CXCL9 or soluble CD14 and plasma HIV RNA, nor with the duration of therapy.

## Discussion

Low-level viremia and viral blips are frequently seen in clinical practice. We observed that an unexpected detectable viral load generates uncertainty among clinicians and patients resulting in a visit to the outpatient clinic ahead of the regular schedule. There is extensive debate whether these patients have an increased risk of virologic failure. Guidelines of DHHS raised the cut-off of virologic failure to 200 copies/mL and stated that patients with isolated blips do not need a change in treatment, whereas for patients with low-level viremia below 200 copies/ml there is no consensus regarding clinical management [11]. Other guidelines continue to use a cut-off of 50 copies/mL for virologic failure [1], [2].

In our cohort, patients with low-level viremia had a higher risk of virologic failure. This corresponds with all five previous studies on this issue [12], [16]–[19] of which four used the cut-off of 50 copies/mL to define low-level viremia [16]–[19]. The most recent large observational study distinguished subgroups based on the level of viremia (50–199, 200–499 and 500–999 copies/mL) and reported an increased risk of virologic failure in all subgroups. Although the risk was highest in patients with viremia between 500–999 copies/mL, patients with low-level viremia between 50 and 200 copies/mL still showed a 2-fold higher risk of failure [12]. In our cohort, two of three patients with virologic failure had low-level viremia between 50 and 200 copies/mL, supporting this observation.

Resistance testing was only performed on request of the physician. Selection of resistance was observed in only 1 of 9 patients, at a VL around 500 copies/mL. Eight out of nine patients were using a boosted PI-based regimen, which may explain the limited selection of resistance as boosted PIs have a high intrinsic genetic barrier for resistance. Previous studies did show selection of resistance, including PI resistance, in a subset of patients with low-level viremia, even at VLs up to 200 copies/mL [20], [21]. The presence of resistance mutations during low-level viremia has been shown to be associated with risk of failure, suggesting that resistance testing has added value in these cases [22], [23]. In a Spanish cohort it was shown that optimization of therapy after resistance testing in patients with low-level viremia resulted in viral suppression below 50 copies/mL in 90% of patients after 1 year [24].

Regarding viral blips, a number of studies have been performed leading to different conclusions. We observed a much lower risk of virologic failure in patients with viral blips compared to patients with low-level viremia. This is in line with nine studies that did not report an increased risk of virologic failure in patients with blips [17], [19], [25]–[31]. As in our cohort, these studies defined a viral blip as a viral load above 50 copies/mL. In contrast, four studies did find an increased risk of virologic failure in patients with viral blips [16], [32]–[34], of which three used a definition of a VL exceeding 400 or 500 copies/mL. A recent study in therapy-naïve patients that distinguished on the level of viral blips (50–199, 200–499 and >500 copies/mL) only reported an increased risk of failure in patients with a blip that exceeded 500 copies/mL [34]. Thus the results of current available literature suggests the height of the blip may be important, explaining why previous literature not taking the viral load into account seemed to be conflicting on this issue.

The nature of blips and low-level viremia is subject of debate and it has been suggested that it is a result of increased sensitivity or assay variation. However, several studies have demonstrated an association between residual viremia measured at a single time point and the risk of viral rebound, using several assays, including ultrasensitive assays and Taqman assay v.2.0 [5], [35]–[37]. Studies that failed to show such an association either had a relatively small sample size or had a rare development of rebound overall [38], [39]. Our cohort is the first that assessed residual viremia in longitudinal samples preceding the viral blip or low-level viremia. We observed that most patients with low-level viremia had residual viremia throughout the preceding year, while more than half of patients in the control group continuously had no detectable viremia. We observed this difference, although to a lesser extent, also for patients who experienced blips. Of interest, the presence of residual viremia was related to the duration of therapy, which has also been shown as a relevant factor in other studies [5], [35], [37].

The association between residual viremia and blips or low-level viremia on one hand and the duration of therapy on the other hand, suggests that we are detecting a biologically relevant phenomenon. With the use of assays with single-copy sensitivity it has been shown that the viral load, after initial decline below 50 copies/mL, continues to decay slowly [3]. Therefore, it is plausible to expect that patients in the first years of therapy more often have residual viremia and are more likely to experience viral blips or low-level viremia. Whether this is a result of virus production or replication remains uncertain and it is not unlikely that both processes coexist. We compared levels of immune activation markers CXCL9 and soluble CD14 and observed slightly higher levels of CXCL9 in patients with low-level viremia. Prospective longitudinal studies are necessary to look further into the possible role of immune activation in residual viremia, viral blips and low-level viremia.

We observed low rates of virologic failure in our cohort. A relevant factor is the active switching of therapy in some patients before virologic failure could occur, which is an inevitable limitation of the retrospective nature of the study. The retrospective design enables selection of all patients who had low-level viremia and viral blips in our clinic. Although the sample size was relatively small, which is reflected in the large confidence intervals in our logistic regression analysis, all values in the interval support the conclusion that residual viremia is associated with the development of low level viremia and viral blips. Based on our results we cannot assess how often patients with residual viremia would develop low-level viremia or viral blips, but it has been shown previously that 10 to 40% of patients with residual viremia will have a rebound above 50 copies/mL [5].

Clinical management of blips and low-level viremia remains a complex issue, as it is difficult to distinguish viral production from viral replication with subsequent risk of selection of resistance. In some cases blips and low-level viremia represents viral production that is irrelevant for long-term virological outcome. Current available observational evidence indicates that patients with blips exceeding 500 copies/mL and patients with persistent viral loads above 50 copies/mL have a higher risk of virologic failure. In these patients it is more likely that viral replication occurred with subsequent risk of selection of drug resistant variants, and therefore a regimen switch after assessing present drug resistance should be highly considered.
